# Supplementary material for: Dynamic enhancer landscapes in human craniofacial development
Source: Nat Commun. 2024 Mar 6;15:2030. doi: 10.1038/s41467-024-46396-4 (PMC10917818; doi:10.1038/s41467-024-46396-4)
Supplement: Supplementary file 1 — Supplementary Information [file 41467_2024_46396_MOESM1_ESM.pdf]

Supplementary Figures

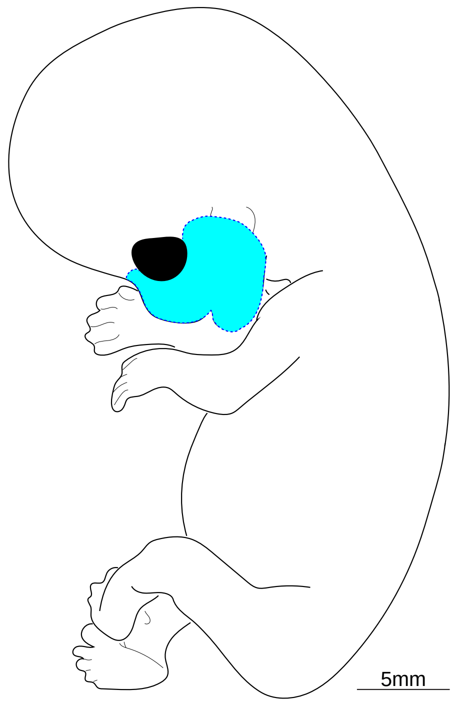

Supplementary Figure 1. Outline of tissue dissection for human face samples received from Human Developmental Biology Resource. Cartoon of a week 8 human embryo showing the outline of the dissected tissues (blue shaded area), identical dissections were performed for all week 7 and 8 embryonic samples (CS18, 19, 22 and 23) received by our laboratory.

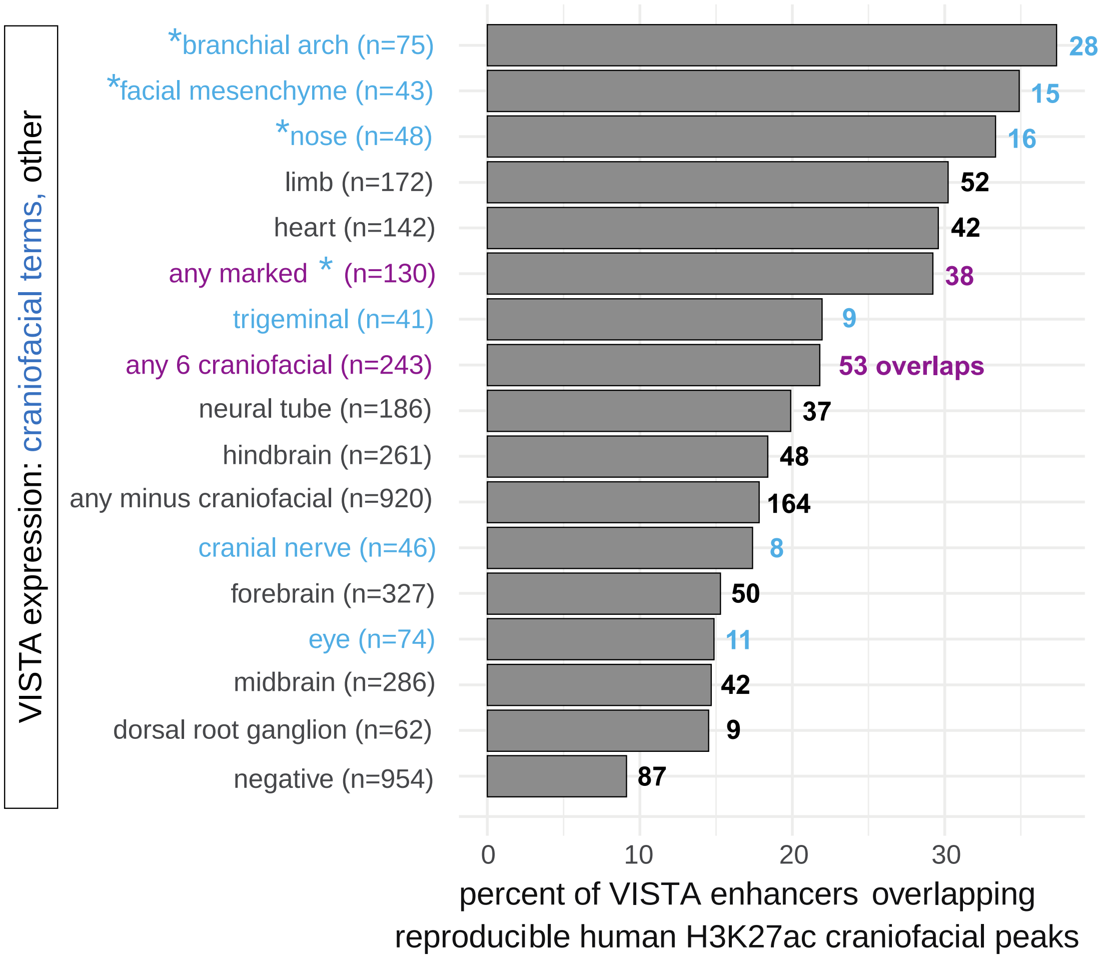

Supplementary Figure 2. Comparison of predicted human face enhancers and VISTA human craniofacial regulatory elements. Y-axis shows terms for recorded expression of enhancer driven *lacZ* for human craniofacial elements reported in the VISTA Enhancer Browser, numbers in parentheses denote the total number (N) of such observations in the VISTA catalog. Specific craniofacial terms are denoted in blue. Grouped terms "any marked*" and "any 6 craniofacial" are shown in purple. Terms for facial mesenchyme, branchial arch and nose are each marked by an asterisk and constitute "any marked*", while "any 6 craniofacial" comprises the six craniofacial terms shown here. Terms with fewer than 40 elements such as those for melanocytes, are not shown individually. Bars on the right indicate the percentage of VISTA enhancers (x-axis) for the relevant expression category that overlap human developmental face enhancers in this study (n=13,983). The number in bold by each bar denotes the absolute number of such overlaps out of the total N for each expression category. See Supplementary Data 4 for a full list of elements positive for craniofacial terms. Source data are provided as a Source Data file.

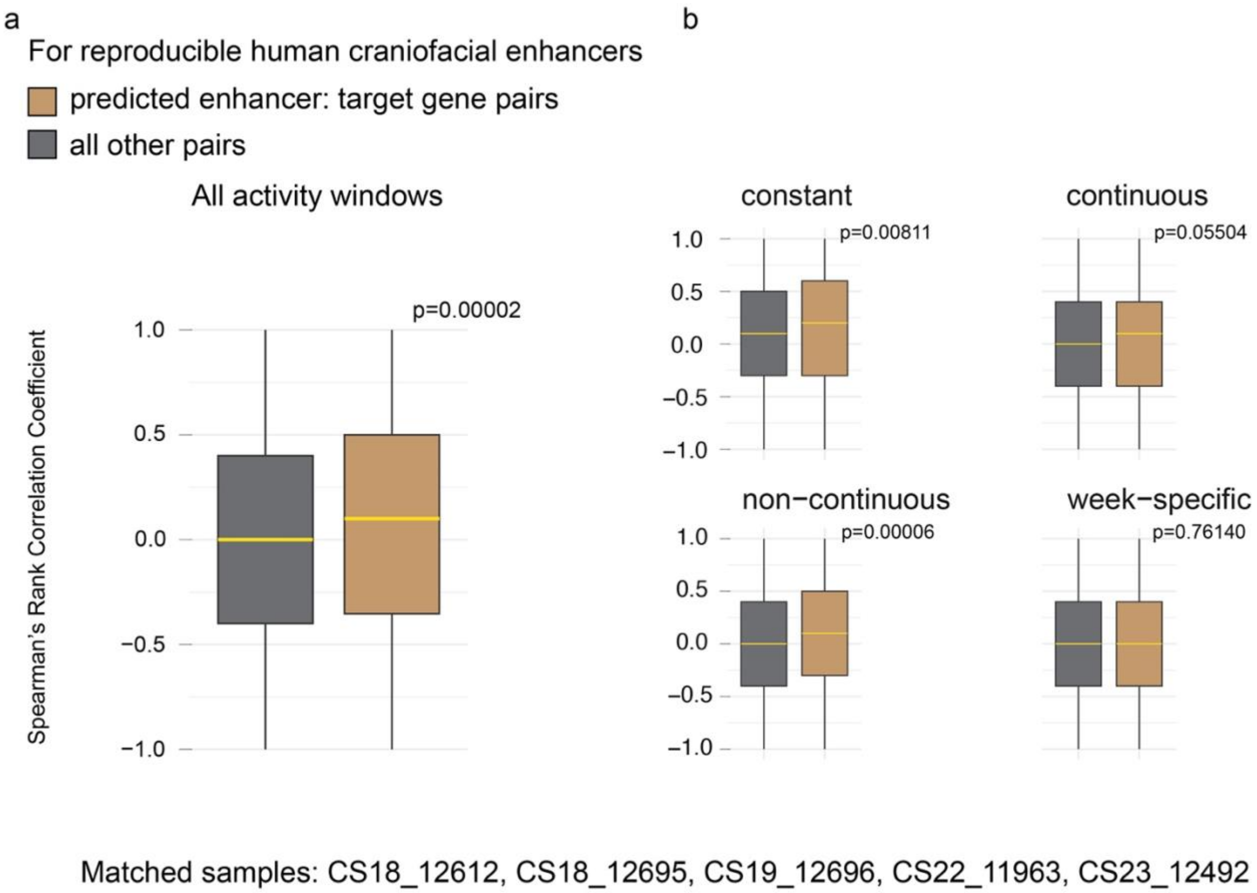

Supplementary Figure 3. Correlations between enhancer activity and gene expression. a/b. Spearman's Ranked Correlation Coefficients were calculated between H3K27ac ChIP-seq signal intensity and gene expression counts for a subset of human reproducible craniofacial enhancers with their assigned target genes based on intersections with promoter-centric long-range interacting fragment predictions from Jung et al., 2019 (see Supplementary Data s 7-8; Methods). Correlations for enhancer:target-gene pairs of interest were compared with all other pairs for human craniofacial enhancers across combined activity windows (**a**) as well as individual activity classes (**b**). Yellow horizontal lines and boxes indicate the median and the 25th/75th percentiles respectively, whiskers indicate the full distribution of the data. p-value: Mann Whitney U. Human embryonic face samples where data for both H3K27ac binding, and gene expression were available from identical samples ("Matched samples" in figure) were used for this analysis. Source data are provided in Source Data file.

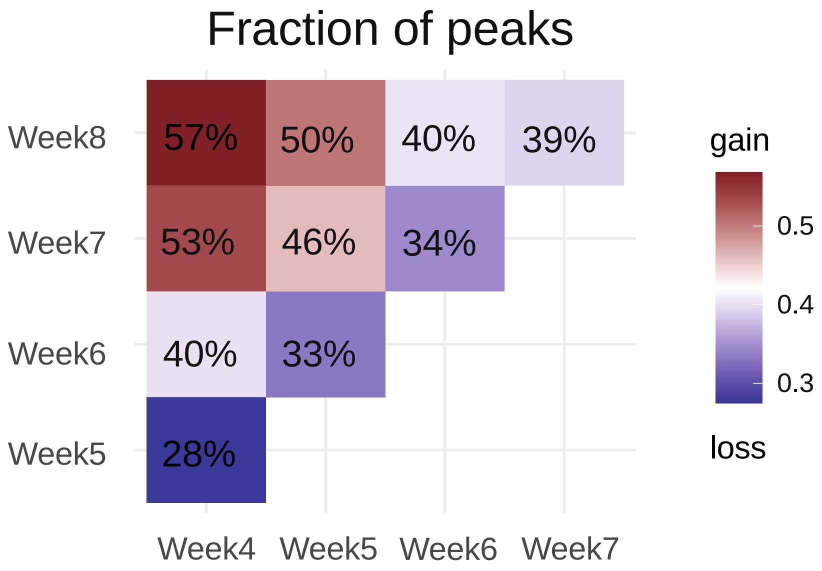

Supplementary Figure 4. Temporal transitions in the number of predicted human enhancer peaks. For the categories of H3K27ac peaks shown in Figure 2b, the fraction of 13, 983 reproducible peaks that change activity between adjacent and nonadjacent weeks of human embryonic craniofacial development are shown for weeks 4-8. See Supplementary Data 2 for annotations of human enhancers by week in development. Source data are provided in Source Data file.

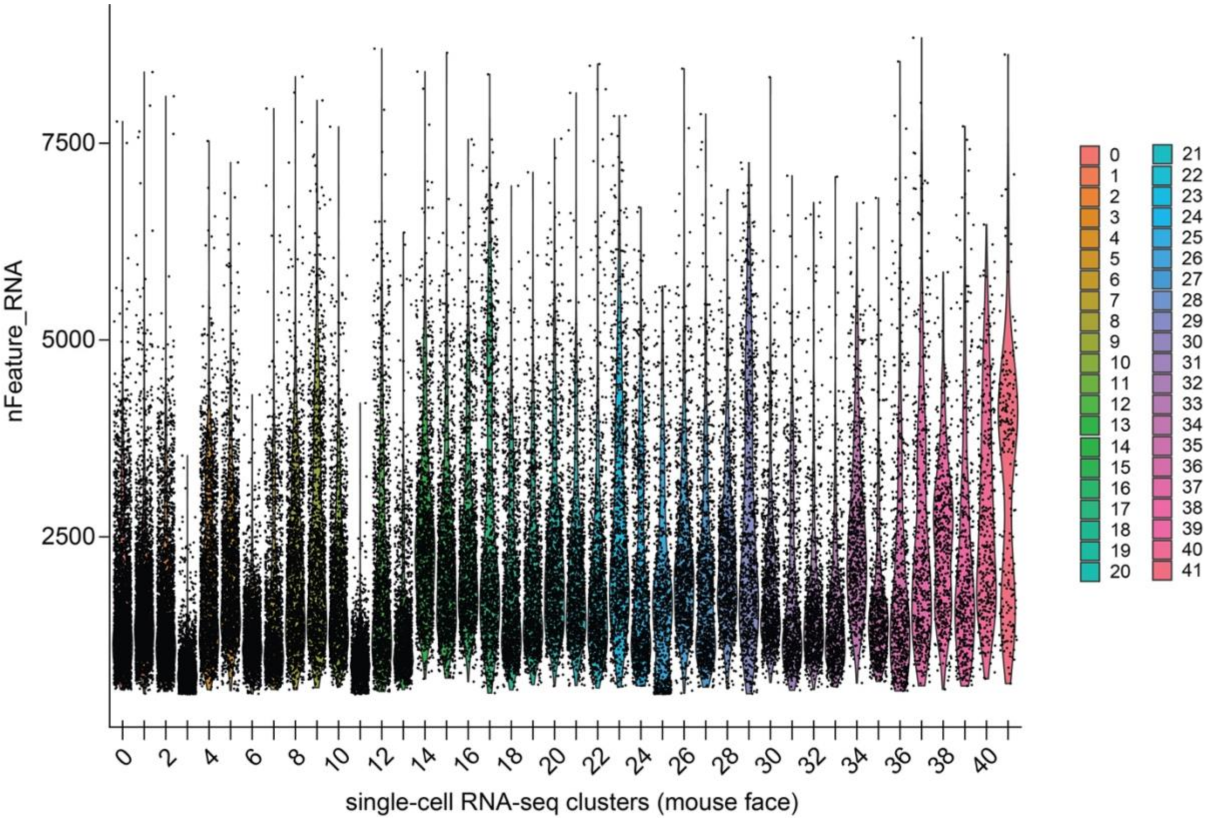

Supplementary Figure 5. Distribution of genes per cell in single-cell gene expression of the mouse face. X-axis shows all detectable clusters in the aggregate of single-cell gene expression from all mouse face samples between e11.5-13.5 processed in this study. y-axis shows the number of genes expressed per cell (nFeature_RNA), after filtering cells with <200 feature counts, >5% mitochondrial transcripts and doublets. Source data are provided as a publicly accessible Seurat/R object file, see Data Availability Statement for details.

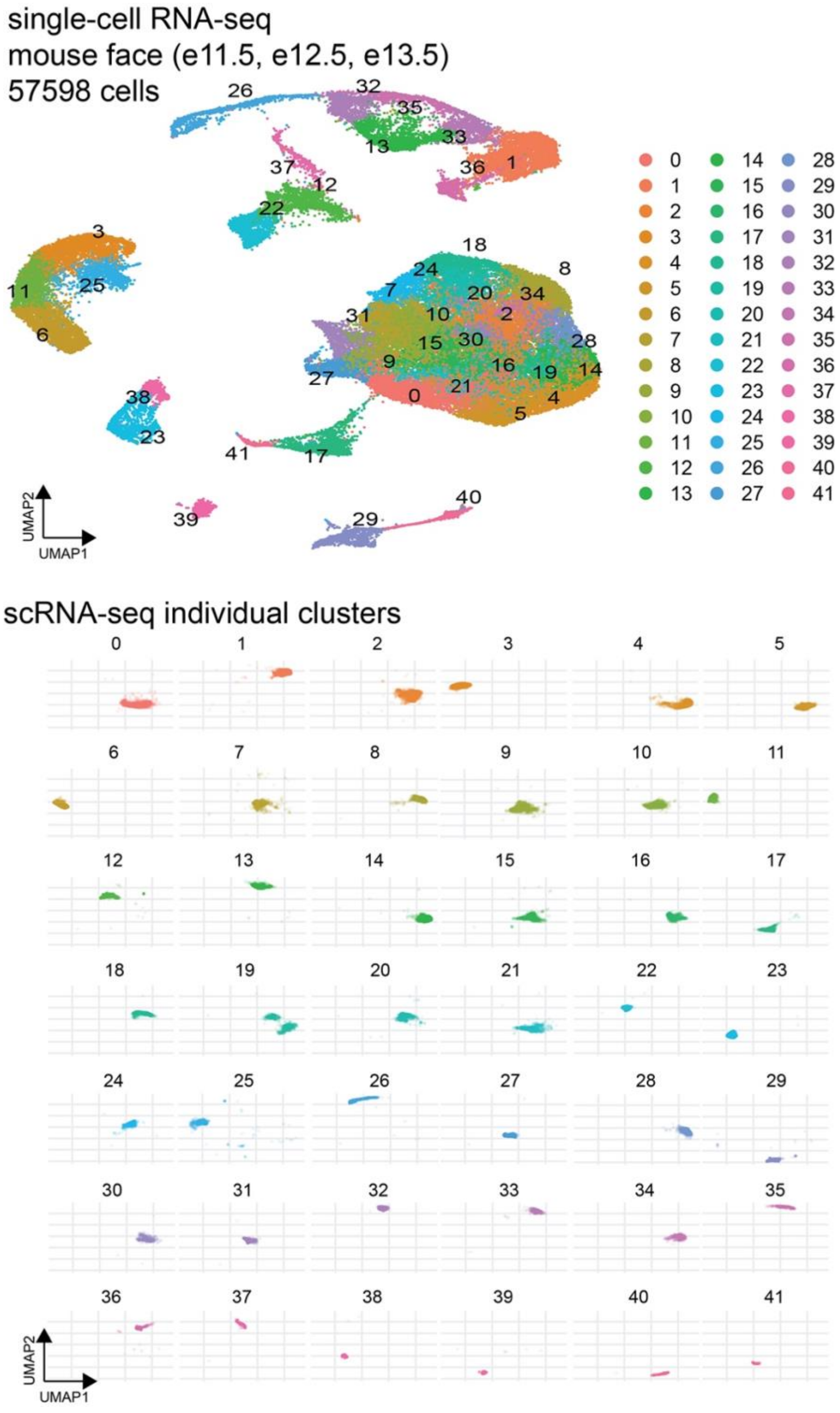

Supplementary Figure 6. Unbiased clustering of single-cell gene expression data of the developing mouse face. For clarity, individual UMAPs are shown to demonstrate the spatial extent along x-y coordinates for each cluster (0-41) that comprises the final UMAP shown in Figure 3. Source data are provided as a publicly accessible Seurat/R object file, see Data Availability Statement for details.

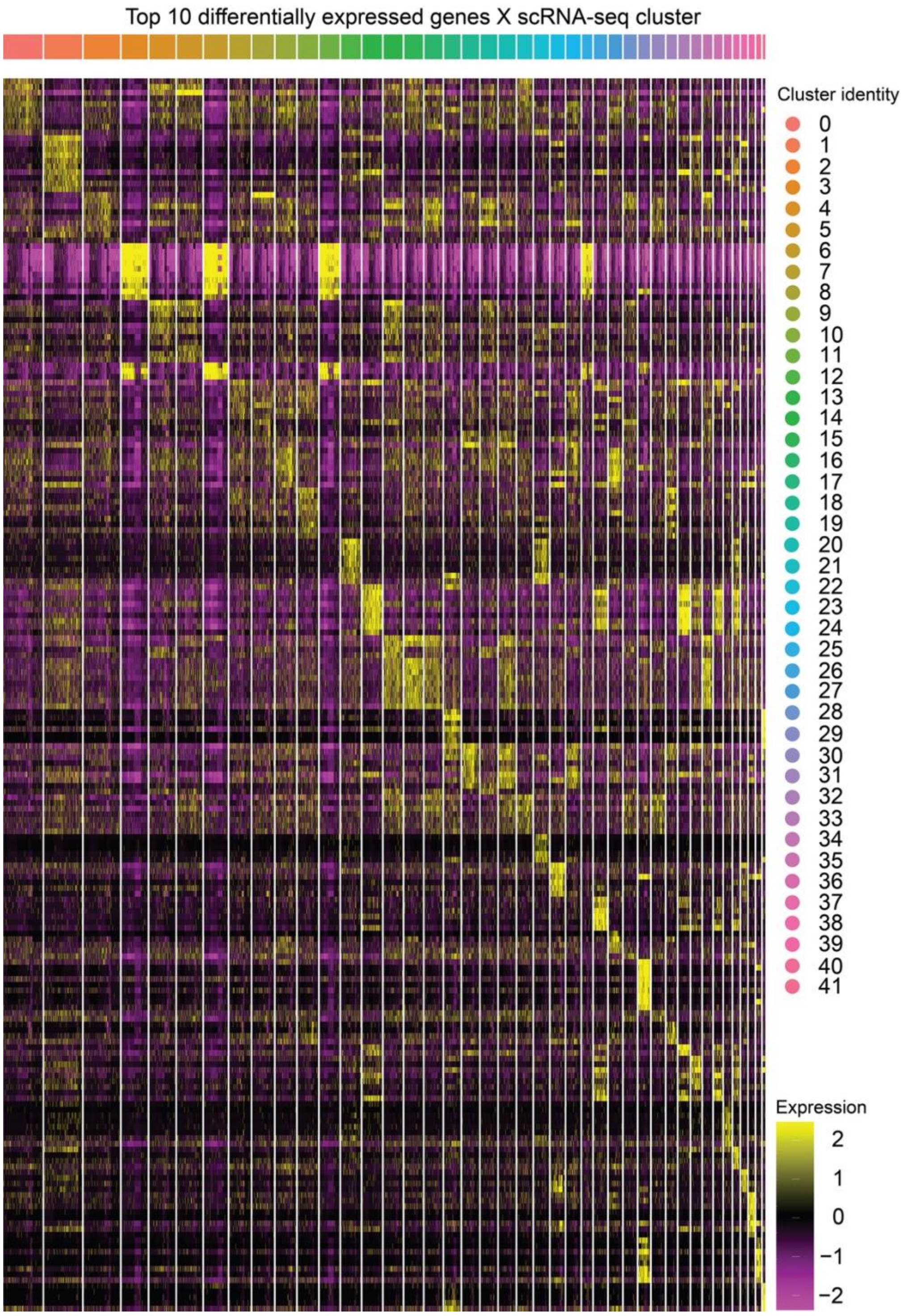

Supplementary Figure 7. Cluster-wise marker genes in single-cell (sc) gene expression data of the developing mouse face. Heatmap shows top 10 marker genes, i.e., genes highly enriched for expression in a cluster over all clusters (y-axis) for each of the 42 original clusters (x-axis) defined in the single-cell gene expression data. See Supplementary Data 14 for top genes by cluster. Source data are provided as a publicly accessible Seurat/R object file, see Data Availability Statement for details.

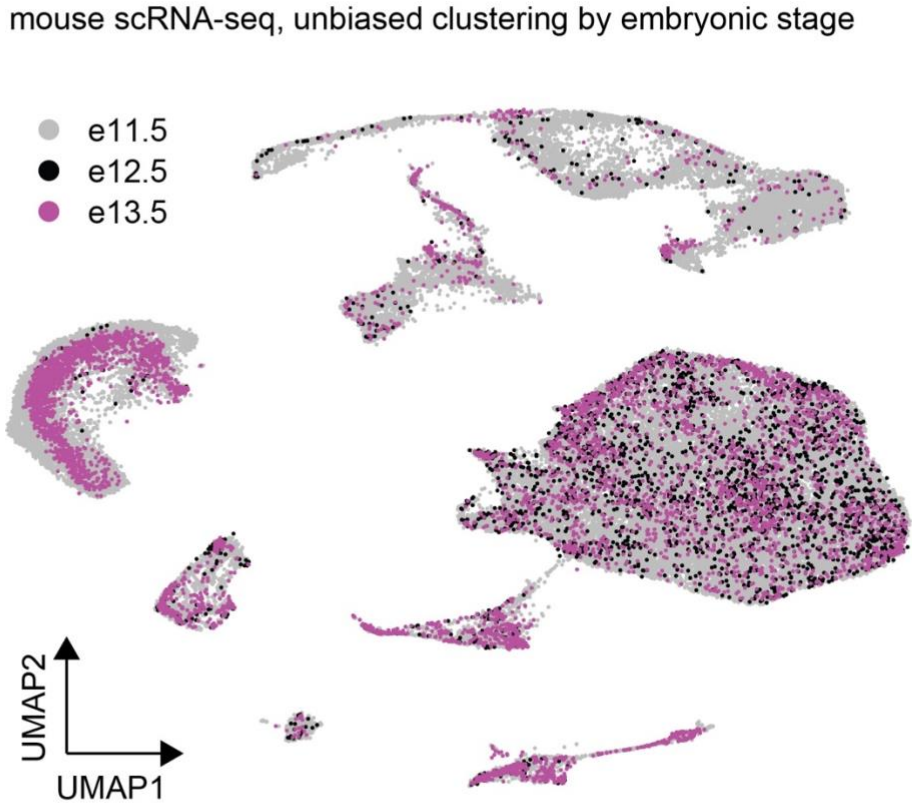

Supplementary Figure 8. Distribution of cells in the single-cell gene expression data by mouse embryonic stage. UMAP shows distribution of cells from respective mouse embryonic stages e11.5 (gray: 49,882 cells), e12.5 (black: 2,340 cells) and e13.5 (magenta: 5,376). Source data are provided as a publicly accessible Seurat/R object file, see Data Availability Statement for details.

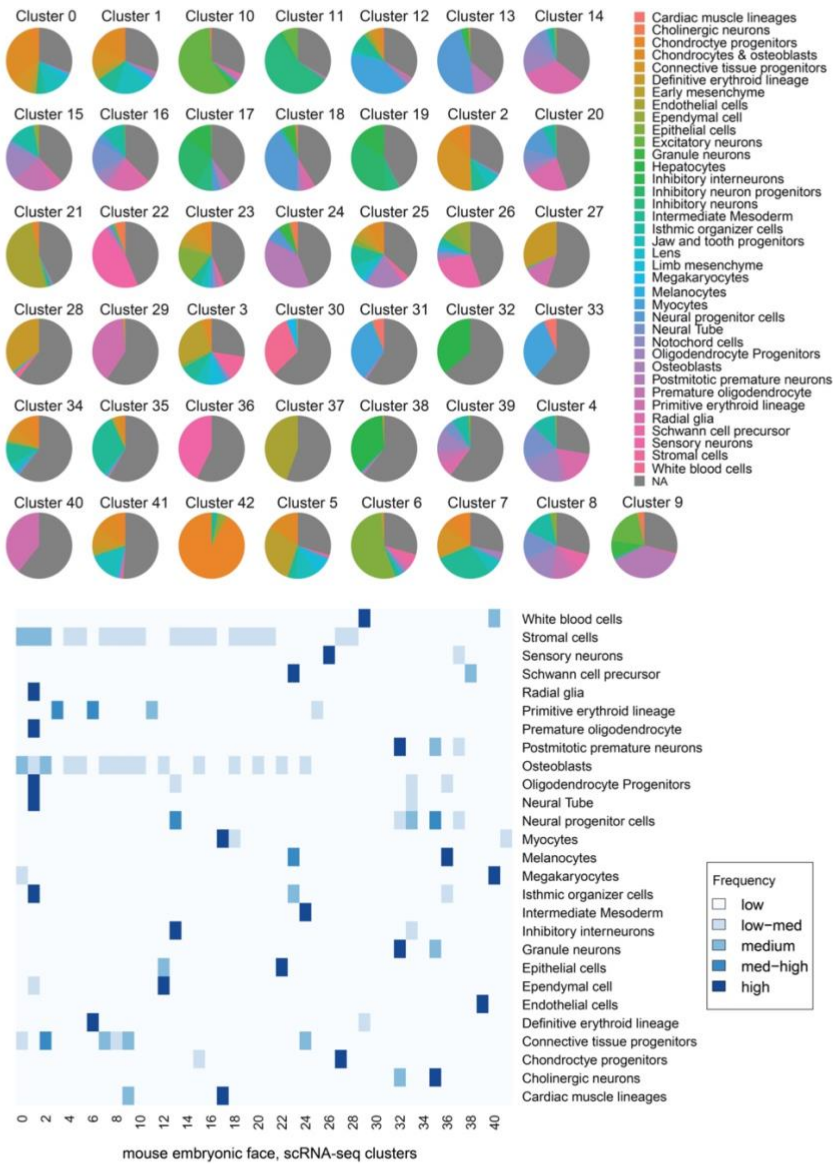

Supplementary Figure 9. Cluster-wise proportion of cell-types in mouse single-cell gene expression data. Our single-cell gene expression data was queried to a previously published scRNA-seq large dataset of whole embryo developmental timepoints (Cao et al., 2019) using Seurat-based auto referencing as a first step for assigning cell-type identities in an unbiased manner. Heatmap shows frequency (low to high) of cell types from the reference (n=27, y-axis) that are reflected in each of the 42 clusters (x-axis). These collectively informed the first broad annotations of cell types for our *ScanFaceX* data. Source data are provided as a publicly accessible Seurat/R object file, see Methods, and Data Availability Statement for details.

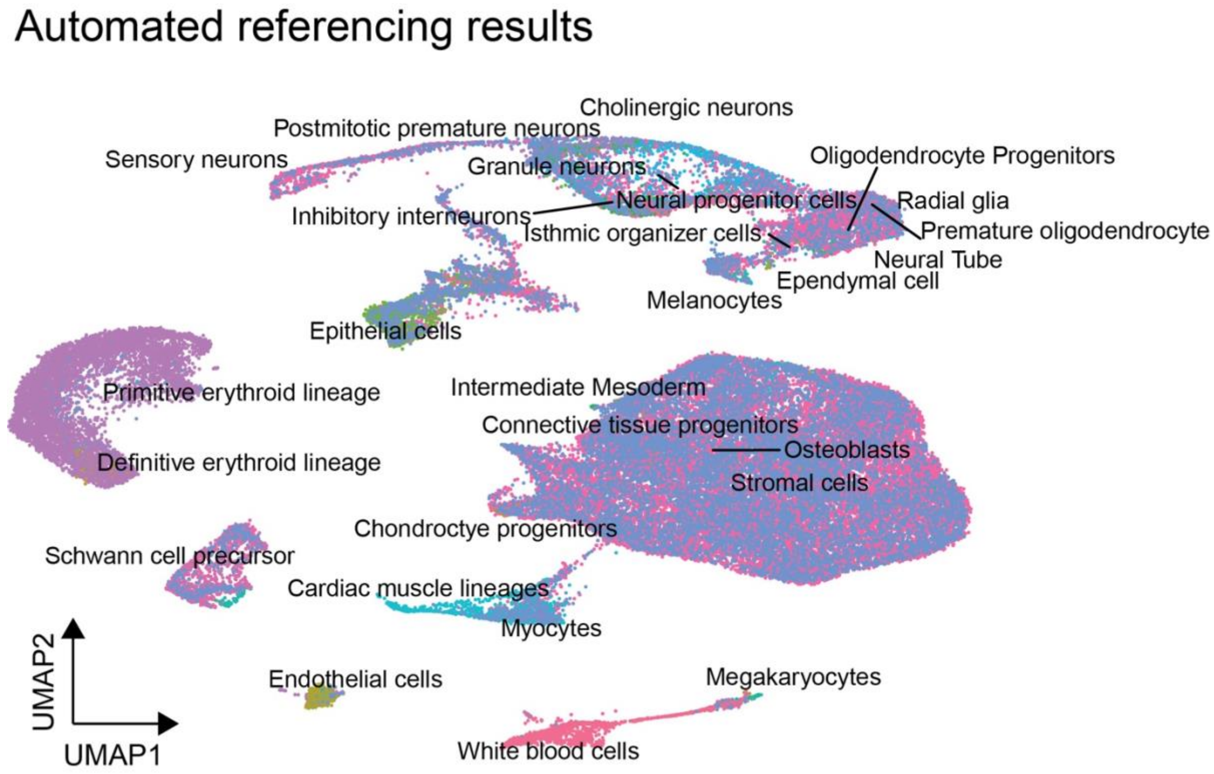

Supplementary Figure 10. Assignment of cell-type identities in *ScanFaceX*. UMAP shows raw results from Seurat-based automated referencing and cell type annotations for *ScanFaceX* data. A down-sampled (100K cells) subset from Cao et al., 2019 whole-embryo single cell data was used as reference. Source data are provided as a publicly accessible Seurat/R object file, see Methods, and Data Availability Statement for details.

Select marker gene expression x scRNA-seq clusters

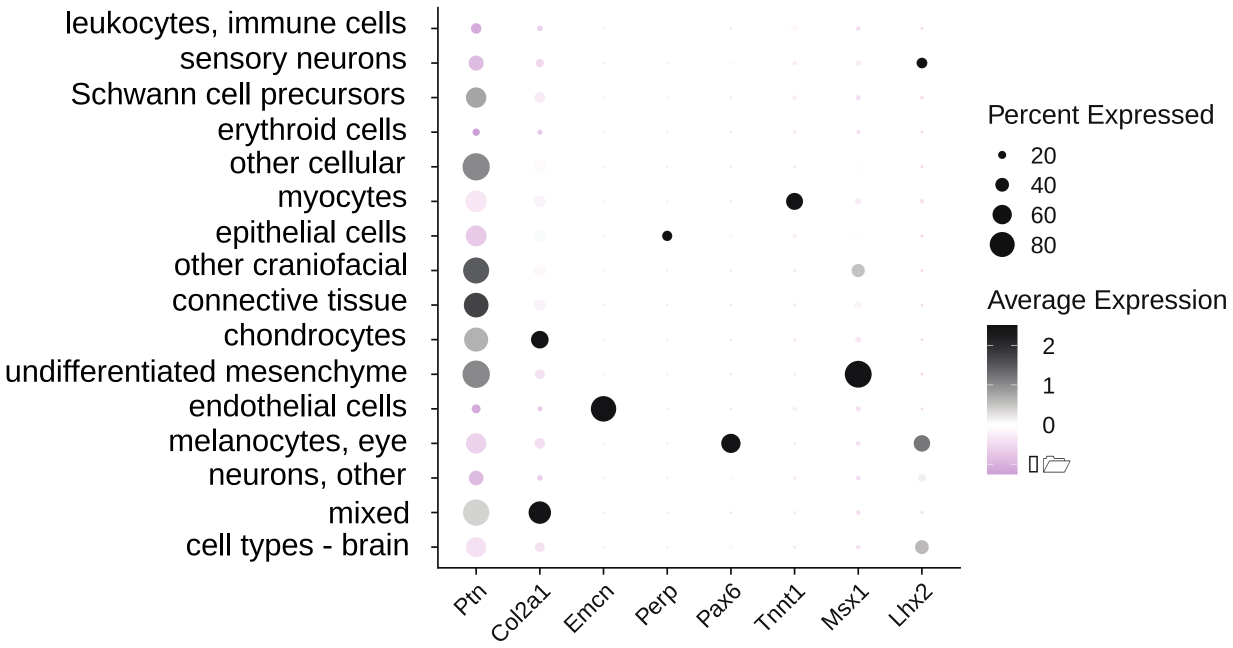

Supplementary Figure 11. Expression of select marker genes across all clusters in *ScanFaceX*. Dot plot shows expression of select marker genes across all original clusters (consolidated per final annotations in Supplementary Data 12) of *ScanFaceX*, a subset of this plot is shown in main Figure 3. Color scale denotes low (light grey) to high (black) expression while increasing circle diameters denote corresponding higher proportion of cells within respective clusters. Source data are provided as a publicly accessible Seurat/R object file, see Data Availability Statement for details.

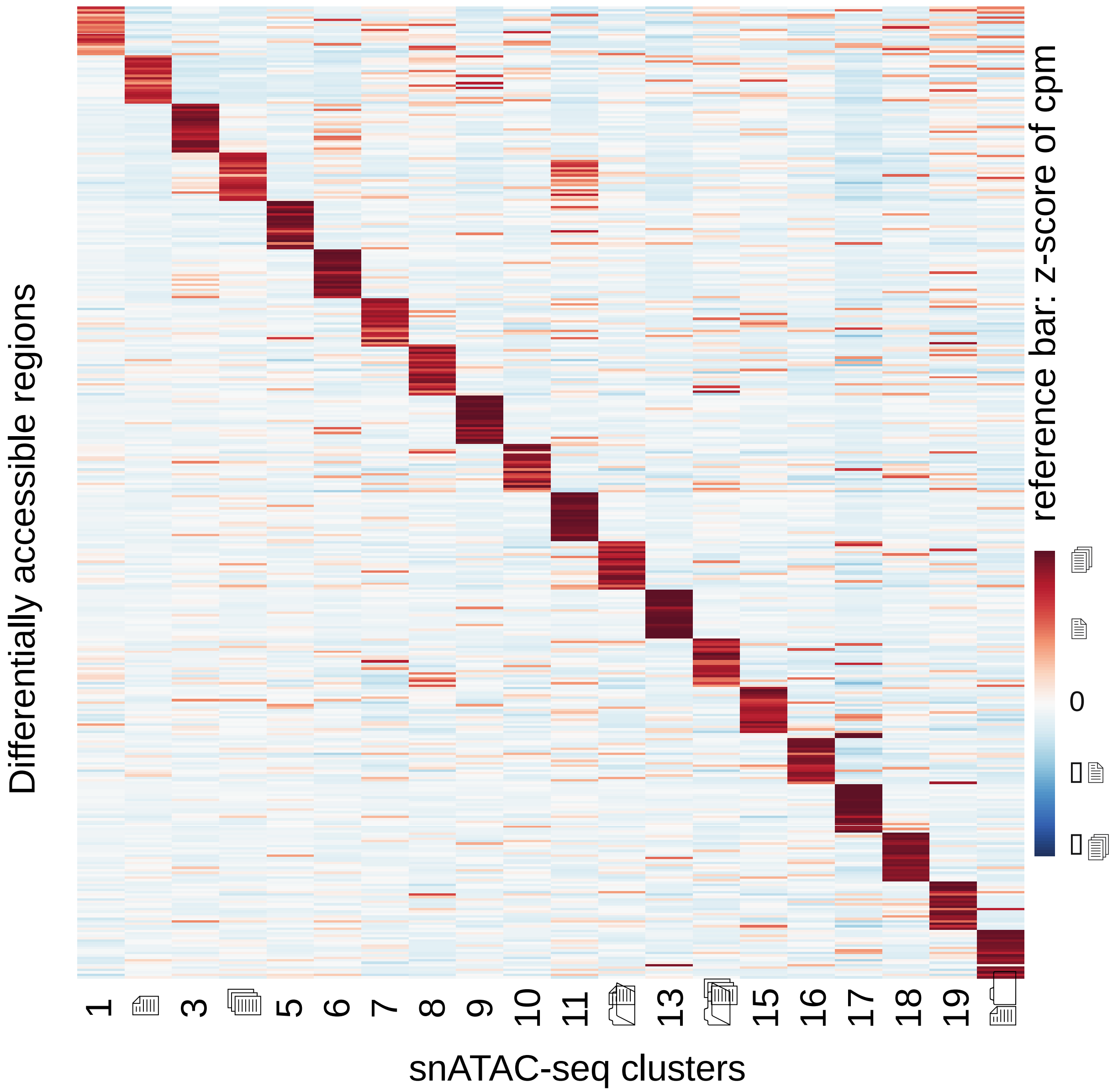

Supplementary Figure 12. Differentially accessible regions in snATAC-seq, mouse face. Heatmap shows the top 20 DARs map exclusively to each of the 20 clusters in snATAC-seq (mouse face). See Supplementary Data 17 for more details. Source data are provided as a publicly accessible Seurat/R object file, see Data Availability Statement for details. Source data are provided in Source Data file.

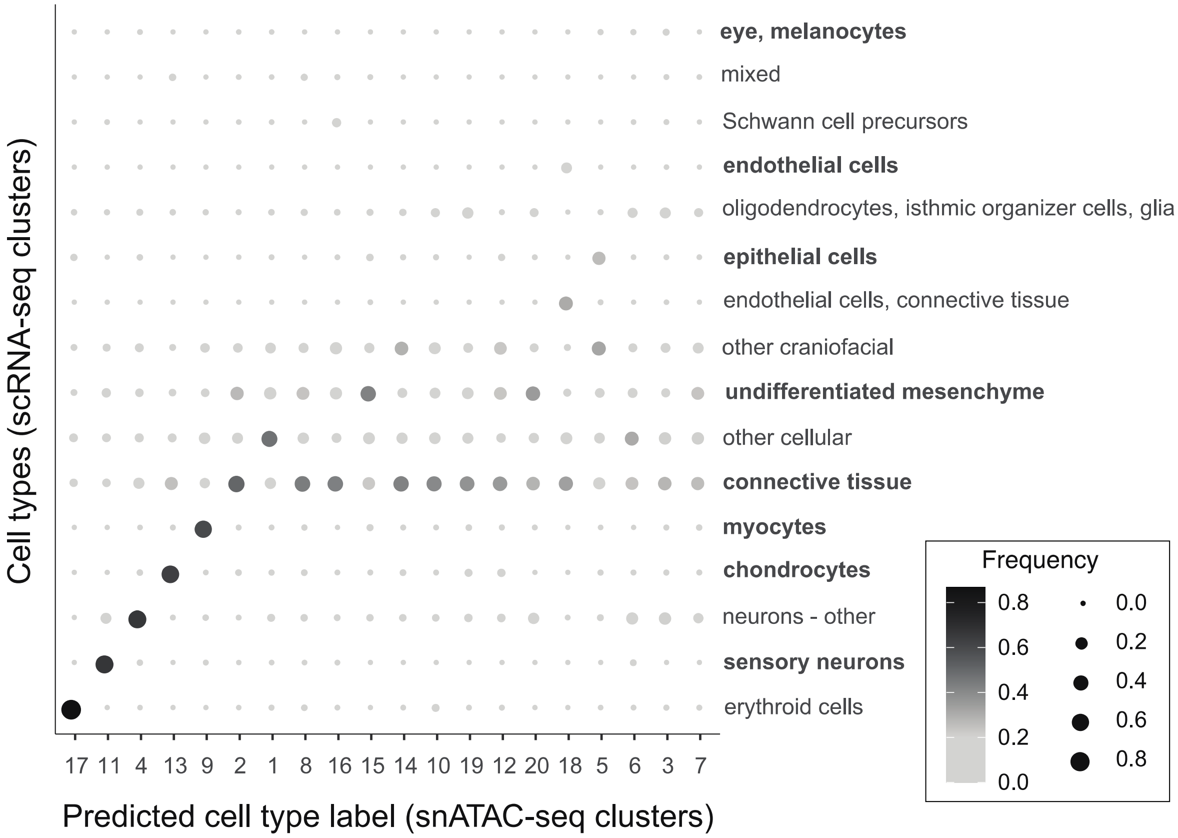

Supplementary Figure 13. Correlation of scRNA-seq and snATAC-seq face data. Dot plot shows correlation, i.e., strength of label transfer between gene expression quantification (scRNA-seq; y-axis; n=16) and accessibility in TSS and gene bodies (snATAC-seq; x-axis; n=20) for integrated gene expression and open chromatin data for final annotated cell-types. Color scale denotes low (light grey) to high (black) degree of correlation while increasing circle diameters denote corresponding higher proportion of cells within correlated cell types for the respective clusters. Cell types in bold are cell types shown in Figures 3-5. Source data are provided in Source Data file.

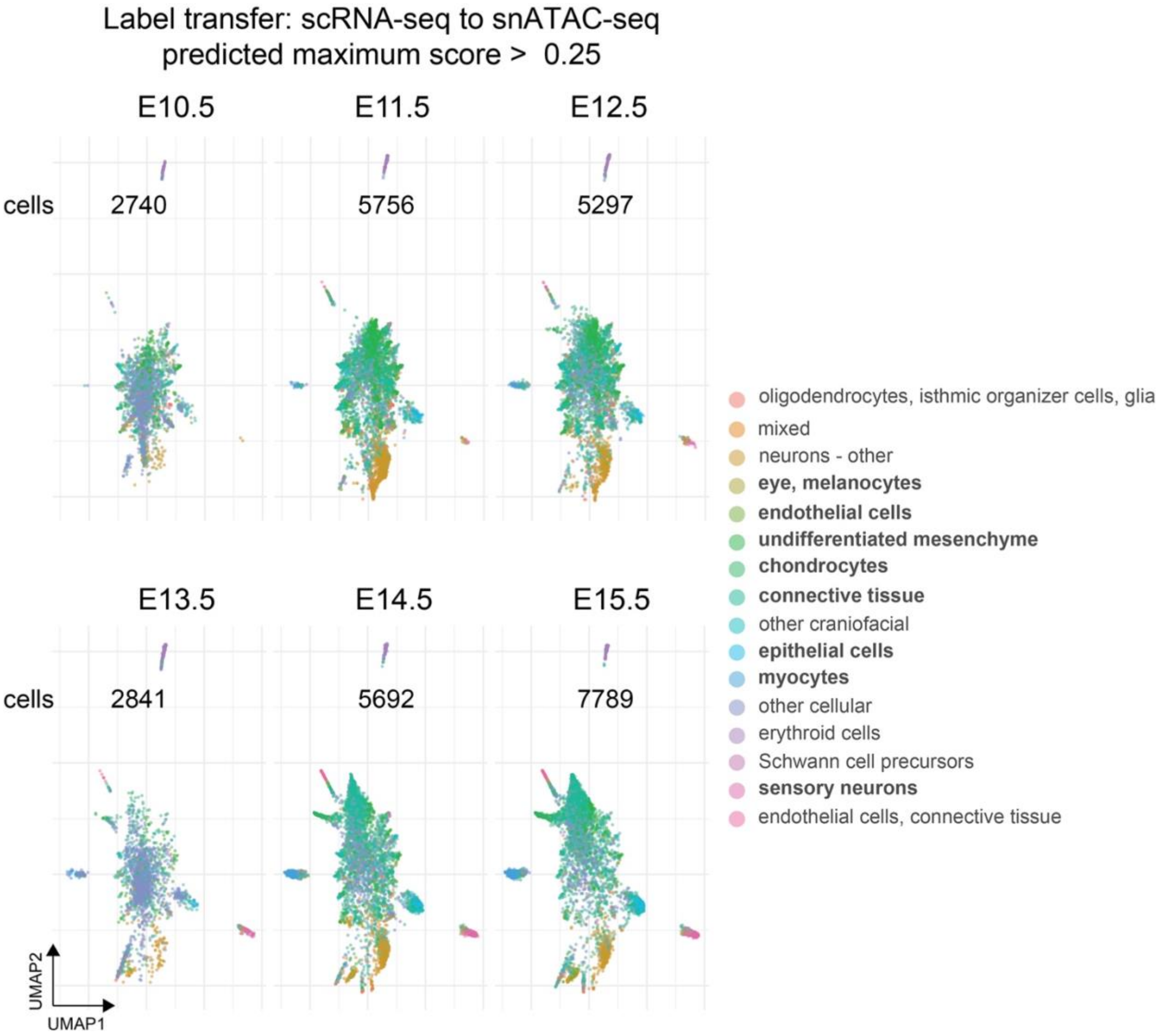

Supplementary Figure 14. Developmental stage-wise correlation of scRNA-seq and snATAC-seq face data. Individual UMAPs show the total number of cells in our snATAC-seq assay that pass the >0.25 threshold for the predicted maximum score for label transfer between the integrated scRNA-seq and snATAC-seq datasets for 16 final cell-type annotations (key on the right). Source data are provided as a Source Data file.

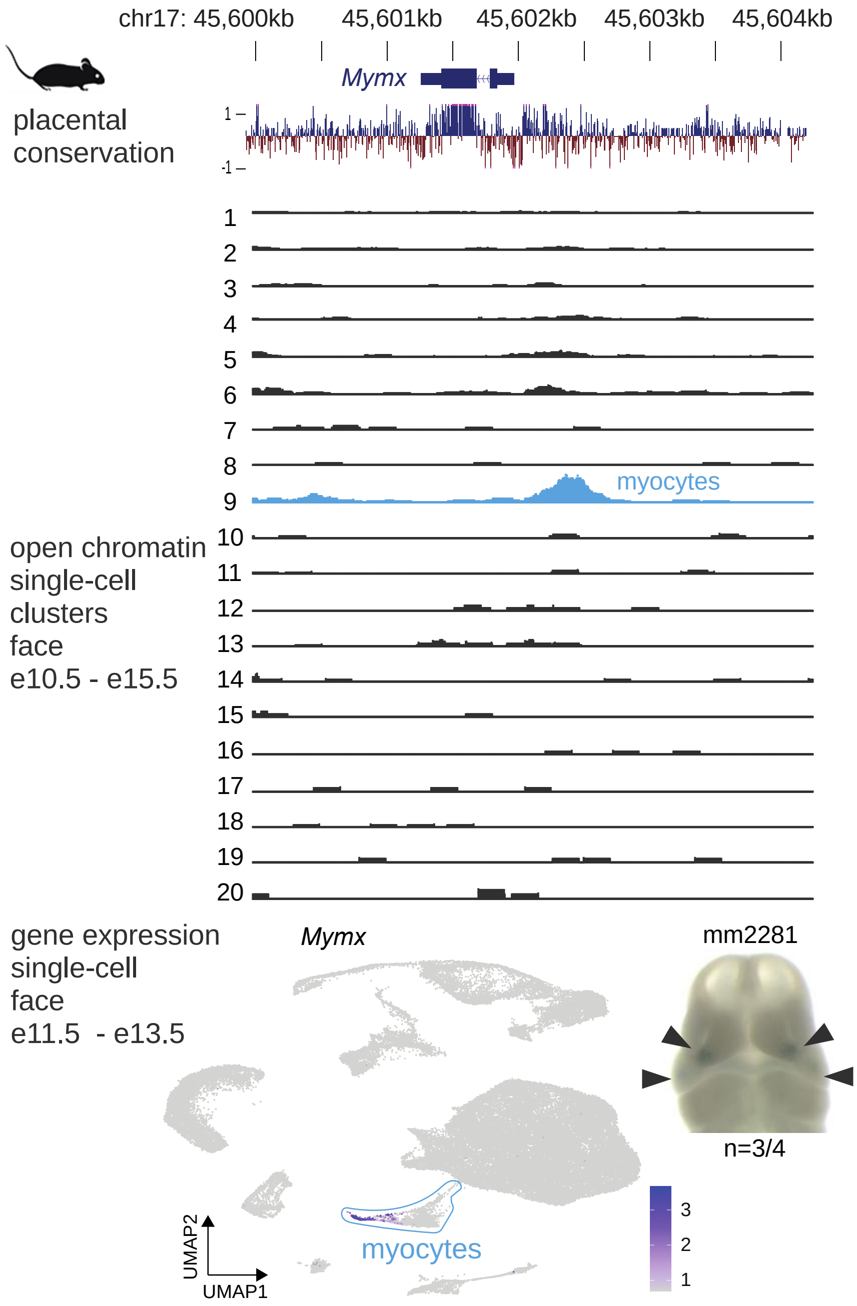

Supplementary Figure 15. Differentially accessible regulatory regions correlate with cell-type specific signatures. The genomic context and placental conservation scores for a regulatory region near *Mymx* promoter are shown, followed by tracks for individual snATAC-seq clusters from developing mouse face tissue (e10.5 – e15.5). This region shows distinct open chromatin signature in the myocyte-specific cluster. UMAP of ScanFaceX shows expression of *Mymx* in myocytes. Image for a representative mouse embryo at e11.5 shows validated *in vivo lacZ*-reporter activity (grey arrowheads) of this enhancer. *MYMX (Myomixer)* encodes an integral membrane protein that regulates myoblast fusion, is conserved across vertebrates and *MYMX* mutations underlie an autosomal recessive disorder, Carey-Fineman-Ziter syndrome-2 (CFZS2) in humans that is characterized by weakness of the facial musculature, hypomimic facies, micrognathia, and facial dysmorphism among a range of other defects. n, reproducibility of each pattern across embryos resulting from independent transgenic integration events.

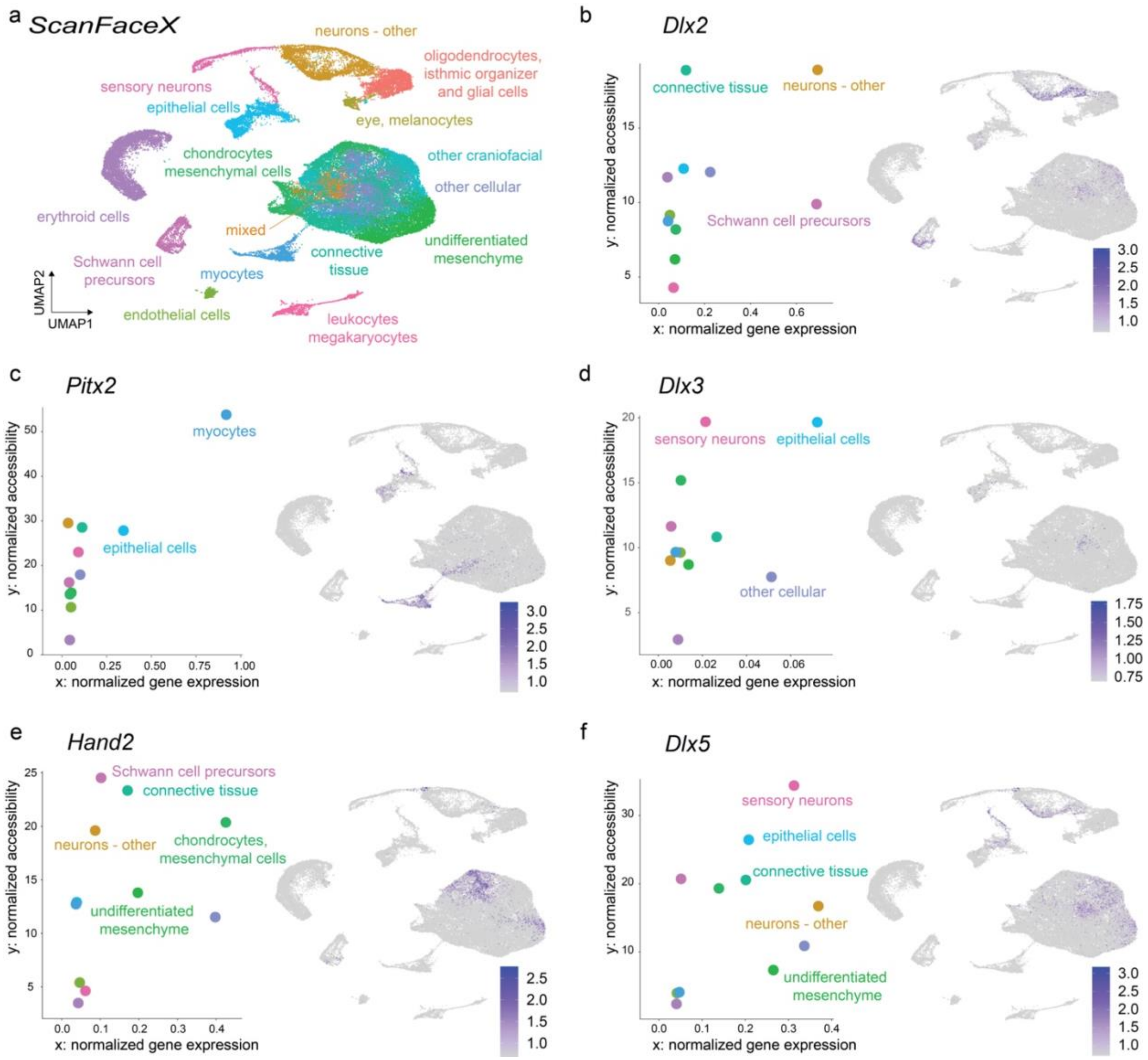

Supplementary Figure 16. Ectomesenchymal marker genes expressed in *ScanFaceX*.a. Uniform Manifold Approximation and Projection (UMAP) clustering, color-coded by inferred cell types in *ScanFaceX* is shown here for ready reference. b-f.Dot plots show correlation between normalized gene expression (x-axis) from *ScanFaceX* and normalized accessibility (y-axis) from *ScanFaceN* for select genes (*Dlx2*, *Pitx2*, *Dlx3*, *Hand2* and *Dlx5*) and their transcription start sites for matched clusters between *ScanFaceX* and *ScanFaceN.* Matched clusters are depicted as colored dots and follow the same color coding as in (a). UMAPs show expression of the respective genes in ScanFaceX. These genes were selected as mouse orthologs of ectomesenchyme markers from single-cell gene expression profiling of embryonic zebrafish (1.5 - 2 days post fertilization) from Fabian et al., 2022. Source data are provided in Source Data file.
