# Supplementary material for: Dynamic enhancer landscapes in human craniofacial development
Source: Nat Commun. 2024 Mar 6;15:2030. doi: 10.1038/s41467-024-46396-4 (PMC10917818; doi:10.1038/s41467-024-46396-4)
Supplement: Supplementary file 3 — Description of Additional Supplementary Files [file 41467_2024_46396_MOESM3_ESM.pdf]

Description of Additional Supplementary Files

Supplementary Data 1. Summary of human embryonic samples. Genome-wide ChIP-seq (H3K27ac), ATAC-seq and RNA-seq data generated and/or presented in this study is shown with details for sample information including embryonic stage, tissue and assay type.

Supplementary Data 2. Distal enhancer peaks (human). Coordinates for chromosome, start and end are shown for 13,983 reproducible peaks across weeks 4-8 of the developing human face for genome-wide anti-H3K27ac ChIP-seq. Coordinates in hg38.

Supplementary Data 3. H3K27ac-bound regions overlapping open chromatin regions. H3K27ac-bound regions (ChIP-seq) from human developing face between weeks 4-8 of embryogenesis that overlap open chromatin regions from corresponding face tissue are shown with genomic coordinates in hg38, along with extent of overlap (last column).

Supplementary Data 4. H3K27ac-bound regions overlapping VISTA craniofacial enhancers. Human enhancers positively identified as craniofacial from the VISTA Enhancer Browser, and annotated for branchial arch, facial mesenchyme, or nose are shown (column A) along with their genomic coordinates in hg38, tissue where positive (column B) along with overlapping H3K27ac-bound regions (ChIP-seq peaks; column C) from human developing face between weeks 4-8 of embryogenesis. Column D shows the VISTA enhancer id for the overlap described. Adjacent key shows descriptions of abbreviated terms used for describing posive tissues (column B) for listed VISTA enhancers.

Supplementary Data 5. Human craniofacial developmental enhancers in this study. The VISTA id, mouse embryonic stage in which the elements were tested *in vivo*, lacZ-reporter acvity status, tissue, genomic coordinates and whether an alternate allele was tested are shown for 60 elements newly tested in this study.

Supplementary Data 6. Top 15 human phenotype terms enriched in our list of 13, 983 reproducible human craniofacial enhancers. For more details, see Methods.

Supplementary Data 7. Enhancer-taget gene predictions. Coordinates for human reproducible enhancers from this study (columns A-C), coordinates for long-range interacting fragments (columns D and E) from capture HiC readouts (Jung et al., 2019), annotated human gene promoters (column F) and extent of base pair overlap (column G) with respective coordinates in column E are summarized for 4,162 successful overlaps.

Supplementary Data 8. Correlations between enhancer activity and gene expression. Human reproducible craniofacial enhancers in this study were assigned target genes based on intersections with promoter-centric long range interacting fragment predictions from Jung et al., 2019 (see Supplemental Table 7, and Methods). Enhancer coordidates, assigned target gene, Spearman's Rank Correlation Coefficient (SRCC), and activity class of craniofacial enhancers is shown for all positively correlated pairs.

Supplementary Data 9. Summary of genome-wide association studies. GWAS compilation from which lead and associated SNPs were obtained for intersecting with predicted human enhancer peaks in this study are shown. Studies described fell under two groups (Normal Variation, and Disease). Publication details shown are downloaded from the NHGRI-EBI Catalog of Genome-wide association studies.

Supplementary Data 10. GWAS SNPs overlapping human developmental craniofacial enhancers. A list of lead and LD SNPs (columns A and B) derived from the relevant population from each of the GWAS studies listed in Supplemental Table 6, GWAS category (column C),

genomic coordinate for the rsid (column D) that was intersected with human developmental craniofacial enhancers (H3K27ac-bound regions; ChIP-seq, column E) and the results of the intersection are shown.

Supplementary Data 11. Distribution of reproducible human enhancer peaks by stage and sample. For each of the post-conception weeks 4-8, the number of H3K27ac peaks and the number of samples from which the data was aggregated are shown.

Supplementary Data 12. Human-mouse conserved enhancers. H3K27ac-bound regions (ChIP-seq) from human developing face between weeks 4-8 of embryogenesis that overlap H3K27ac-bound regions from mouse embryonic face are shown with genomic coordinates in hg38, along with extent of overlap (last column).

Supplementary Data 13. Summary of mouse single-cell datasets. All mouse face samples along with their embryonic stages that are part of *ScanFace X* as well as *ScanFaceN* along with assay type are listed.

Supplementary Data 14. Top 20 markers for single-cell gene expression for each of the clusters (mouse embryonic face tissue). Key shows the description for the abbreviations in column headers for the table on the left.

Supplementary Data 15. Initial annotations for cell-types in *ScanFaceX*. Broad cell-type annotations as derived from auto-referencing (see Methods) from two different methods are shown with their resulting output for respective clusters.

Supplementary Data 16. Cell type annotations for *ScanFaceX*.

Supplementary Data 17. Differentially Accessible Regions (DARs) for snATAC-seq: mouse face. Genomic coordinates and enrichment for DARs along with p-values and FDR, for respective cluster numbers are shown. Differential accessible region analysis was performed using the SnapATAC 'findDAR' function, which uses the edgeR(v3.18.1) implementation of a one-sided exact test with BCV=0.1. P-value was adjusted to False Discovery Rate (FDR) using Benjamini-Hochberg correction. Last column indicates whether the cluster matched single-cell gene expression clusters from mouse face.

Supplementary Data 18. *ScanFaceN* - all 115, 521 regions.

Supplementary Data 19. *ScanFaceN* - select regions. 7,899 human tissue-derived facial candidate enhancers overlapping with an accessible chromatin region in the *ScanFaceN* catalog.

Supplementary Data 20. Top 50 markers for single-nucleus ATAC-seq for each of the clusters for mouse embryonic face tissue. Analysis was performed using the SnapATAC 'findDAR' function, which uses the edgeR(v3.18.1) implementation of a one-sided exact test with BCV=0.1. P-value was adjusted to False Discovery Rate (FDR) using Benjamini-Hochberg correction.

Supplementary Data 21. Summary of numbers of reproducible human enhancers in this study and corresponding overlaps with relevant lists of enhancers from published literature. For the list of 13, 983 human craniofacial enhancers (H3K27ac peaks), overlaps with enhancers identified from single-cell experiments in this study and overlaps with relevant enhancer lists from previously published data are shown.

Supplementary Data 22. Genomic coordinates of VISTA enhancers (mouse) described in main text and extended data.
