# Supplementary material for: Dynamic enhancer landscapes in human craniofacial development
Source: Nat Commun. 2024 Mar 6;15:2030. doi: 10.1038/s41467-024-46396-4 (PMC10917818; doi:10.1038/s41467-024-46396-4)
Supplement: Supplementary file 5 — Reporting Summary [file 41467_2024_46396_MOESM5_ESM.pdf]

nature portfolio

Corresponding author(s): Axel Visel

Last updated by author(s): 2024/01/26

Reporting Summary

Nature Portfolio wishes to improve the reproducibility of the work that we publish. This form provides structure for consistency and transparency in reporting. For further information on Nature Portfolio policies, see our Editorial Policies and the Editorial Policy Checklist.

Statistics

For all statistical analyses, confirm that the following items are present in the figure legend, table legend, main text, or Methods section.

n/a	Confirmed	
☐	☒	The exact sample size (*n*) for each experimental group/condition, given as a discrete number and unit of measurement
☐	☒	A statement on whether measurements were taken from distinct samples or whether the same sample was measured repeatedly
☐	☒	The statistical test(s) used AND whether they are one- or two-sided<br>*Only common tests should be described solely by name; describe more complex techniques in the Methods section.*
☐	☒	A description of all covariates tested
☐	☒	A description of any assumptions or corrections, such as tests of normality and adjustment for multiple comparisons
☐	☒	A full description of the statistical parameters including central tendency (e.g. means) or other basic estimates (e.g. regression coefficient) AND variation (e.g. standard deviation) or associated estimates of uncertainty (e.g. confidence intervals)
☐	☒	For null hypothesis testing, the test statistic (e.g. *F*, *t*, *r*) with confidence intervals, effect sizes, degrees of freedom and *P* value noted<br>*Give P values as exact values whenever suitable.*
☒	☐	For Bayesian analysis, information on the choice of priors and Markov chain Monte Carlo settings
☐	☒	For hierarchical and complex designs, identification of the appropriate level for tests and full reporting of outcomes
☐	☒	Estimates of effect sizes (e.g. Cohen's *d*, Pearson's *r*), indicating how they were calculated

Our web collection on statistics for biologists contains articles on many of the points above.

Software and code

Policy information about availability of computer code

Data collection	No software was used for data collection
Data analysis	For ChIP-seq, BWA v0.7.7 was used for mapping to the genome, samtools v0.1.19 was used for generating sorted bam files and peaks were called using MACS2 v2.2.4. For bulk face ATAC-seq, Bowtie2 was used for aligning and filtering reads. For bulk face RNA-seq, STAR align V2.12 was used to reference mapping, bamsignals v2.2.1 was used to generate genome wide coverage plots and RSEM v1.4.1for gene expression counts along with GENCODE v24 (version hg38) and GENCODE M4 (version mm10) for gene annotations. rGREAT ontology analyses was performed with rGREAT Bioconductor version: Release 3.17.<br>SNiPA tool (http://snipa.helmholtz-muenchen.de/snipa/) was used for obtaining SNPs in LD for for the lead SNPs reported in the referenced GWAS data. Single-cell gene expression experiments were performed on the 10X Genomics Chromium platform, raw sequence files and feature-barcode matrices were generated with Cell Ranger 3.1.0 and downstream analyses and visualization was performed following Seurat v3.2 and scoreMarkers from scran Bioconductor version: 3.13. snapATAC2 version 1 was used for counting reads and clustering of the snATAC-seq data. Data was analyzed, statistics were estimated and results were generated using existing packages in the statistical computing environment R (www.r-project.org)/ R version 4.1.0. No custom algorithms or software were used.

For manuscripts utilizing custom algorithms or software that are central to the research but not yet described in published literature, software must be made available to editors and reviewers. We strongly encourage code deposition in a community repository (e.g. GitHub). See the Nature Portfolio guidelines for submitting code & software for further information.

Data

Policy information about availability of data

All manuscripts must include a data availability statement. This statement should provide the following information, where applicable:

- Accession codes, unique identifiers, or web links for publicly available datasets
- A description of any restrictions on data availability
- For clinical datasets or third party data, please ensure that the statement adheres to our policy

Wherever applicable, reference genomes Human GRCh38/hg38 and Mouse GRCm38/mm10 were used for alignment and comparisons. The ChIP-seq, ATAC-seq, RNA-seq as well as scRNA-seq and snATAC-seq data presented in this publication, and generated as part of this study are accessible at the National Institute of Dental and Craniofacial Research's FaceBase82,111,133,134 Consortium (facebase.org), and can be found under the following records: RNA-seq, ChIP-seq and ATAC-seq analysis of human fetal tissue, FaceBase Consortium Accession: FB00001358 https://doi.org/10.25550/3C-4G62. Single-cell RNA-seq and single-nucleus ATAC-seq analysis of mouse embryonic tissue, FaceBase Consortium Accession: FB00001359 https://doi.org/10.25550/3C-4R98. These data are additionally deposited in NCBI's Gene Expression Omnibus135,136and are accessible through GEO Series Accession GSE235858 https://www.ncbi.nlm.nih.gov/geo/query/acc.cgi?acc=GSE235858. Other published datasets used in the analyses are described in detail in Methods, cited and listed in Supplementary Data. The NHGRI-EBI Catalog of Genome-wide association studies is accessible at https://www.ebi.ac.uk/gwas/home, and the FANTOM5 database is accessible at https://fantom.gsc.riken.jp/5/. Images of embryos with lacZ-reporter activity are available from the VISTA Enhancer Browser https://enhancer.lbl.gov/. Source data are provided in the Source Data File with this paper, and as publicly accessible Seurat/R objects as applicable.

Research involving human participants, their data, or biological material

Policy information about studies with human participants or human data. See also policy information about sex, gender (identity/presentation), and sexual orientation and race, ethnicity and racism.

Reporting on sex and gender

Embryos of both sexes were included in the experiments. However, we did not consider embryo sex as a variable in our studies since craniofacial development is expected to show minimal differences at these early stages of development.

Reporting on race, ethnicity, or other socially relevant groupings

n/a

Population characteristics

Human embryonic face samples between post-conception weeks 7-8 were used to generate the primary data reported in this study. Karyotype information of the samples is as follows:
CS18_12612 46, XX
CS18_12676 46, XX
CS18_12695 46, XY
CS19_12696 46, XY
CS22_11963 46, XX
CS22_12523r1 46, XY
CS22_12523r2 46, XY
CS23_12492 46, XY
CS18_12676 46, XX
CS18_12695 46, XY
CS19_12696 46, XY
CS22_12498 46, XY
CS23_12492 46, XY
CS18_11904 46, XY
CS18_12041 46, XX
CS18_12612 46, XX
CS18_12695 46, XY
CS19_12696 46, XY
CS22_11865 46, XY
CS22_11940 46, XY
CS22_11963 46, XX
CS22_12425 46, XY
CS22_12493 46, XX
CS22_12498 46, XY
CS22_12706 46, XY
CS23_11846 46, XX
CS23_11884 46, XX
CS23_12440 46, XX
CS23_12492 46, XY

Recruitment

n/a

Ethics oversight

All aspects involving human tissue samples were reviewed and approved by the Human Subjects Committee at Lawrence Berkeley National Laboratory (LBNL) Protocol Nos. 00023126 and 00022756.

Human embryonic face samples were obtained from the Human Developmental Biology Resource's Newcastle site (HDBR, hdbr.org), in compliance with applicable state and federal laws. The National Research Ethics Service reviewed the HDBR study under REC Ref 23/NE/0135, and IRAS project ID: 330783 in compliance with requirements from the National Health Services for research within the UK and overseas. HDBR is a non-commercial entity funded by the Wellcome Trust and Medical Research Council. Fetal tissue donation is confidential, anonymized, completely voluntary with fully informed and explicitly documented written consent, and the participants do not receive compensation. In accordance, no identifying information for human samples in this study was shared by HDBR. More information about HDBR policies and ethical approvals can be accessed at https://www.hdbr.org/ethical-approvals.

Note that full information on the approval of the study protocol must also be provided in the manuscript.

Field-specific reporting

Please select the one below that is the best fit for your research. If you are not sure, read the appropriate sections before making your selection.

[x] Life sciences [ ] Behavioural & social sciences [ ] Ecological, evolutionary & environmental sciences

For a reference copy of the document with all sections, see nature.com/documents/nr-reporting-summary-flat.pdf

Life sciences study design

All studies must disclose on these points even when the disclosure is negative.

Sample size: Details are provided in the Methods section, also where specific information is related to the experimental approach used.

Data exclusions: No data was excluded from the study except where well-established criteria determined specific data filtering steps.

Replication: For human embryonic face samples, we performed experiments with biological replicates as follows: three at CS18, one at CS19, two at CS22 (with two technical replicates for one of two samples), one at CS19 for ChIP-seq. We performed experiments with two biological replicates at CS18, and one each at CS19, and CS22-23 for ATAC-seq; four replicates at CS18, one at CS19, seven at CS22, and four at CS23 for RNA-seq. For single-cell experiments of the mouse face, we performed experiments for eight biological replicates at E11.5, and four replicates each at E12.5 and E13.5 respectively for scRNA-seq, while single samples at each of the six mouse embryonic stages (E10.5, E11.5, E12.5, E13.5, E14.5, and E15.5) were processed for snATAC-seq. For transgenic assays primarily performed and reported in this study, we confirmed results in at least two independent animals (range 2-10 positive results) and used criteria consistent with our site-directed transgenesis pipeline established for the VISTA Enhancer Browser. Results were consistent for experiments with replicates.

Randomization: The experiments were not randomized, not relevant given the study is of observational nature.

Blinding: Individuals who qualitatively assessed the results of in vivo transgenic reporter assays were blinded to genotyping information. For all other experiments, the investigators were not blinded to allocation during experiments and outcome assessment as it was not directly relevant.

Reporting for specific materials, systems and methods

We require information from authors about some types of materials, experimental systems and methods used in many studies. Here, indicate whether each material, system or method listed is relevant to your study. If you are not sure if a list item applies to your research, read the appropriate section before selecting a response.

Materials & experimental systems

n/a	Involved in the study	
[ ]	[x]	Antibodies
[ ]	[x]	Eukaryotic cell lines
[x]	[ ]	Palaeontology and archaeology
[ ]	[x]	Animals and other organisms
[x]	[ ]	Clinical data
[x]	[ ]	Dual use research of concern
[x]	[ ]	Plants

Methods

n/a	Involved in the study	
[ ]	[x]	ChIP-seq
[x]	[ ]	Flow cytometry
[x]	[ ]	MRI-based neuroimaging

Antibodies

Antibodies used: Anti-H3K27ac antibody, Active Motif, Cat# 39133, Lot 01613007.

Validation: Extensively validated for human.

Eukaryotic cell lines

Policy information about cell lines and Sex and Gender in Research

Cell line source(s)	Human Embryonic Kidney 293T/17 [HEK 293T/17] cell line was obtained from ATCC no. CRL-11268.
Authentication	The cell line was purchased from reliable and authenticated vendors. The expected epithelial cell morphology was observed and confirmed visually.
Mycoplasma contamination	The cell lines were not tested for Mycoplasma contamination.<br>We note that we only used this cell line as a generic human DNA spike-in for sequencing libraries. For this purpose, the exact identity of the cell line used, as well as possible presence of mycoplasma DNA, would have no impact on our results, since only sequence reads mapping to the mouse genome were used in our analysis.
Commonly misidentified lines<br>(See ICLAC register)	The cell lines used are not listed in the commonly misidentified lines in the ICLAC register.

Animals and other research organisms

Policy information about studies involving animals; ARRIVE guidelines recommended for reporting animal research, and Sex and Gender in Research

Laboratory animals	Mice used for this study were housed at the LBNL Animal Care Facility, which is fully accredited by AAALAC International. Mice were housed on a 12-hour light-dark cycle in standard micro-isolator cages on hardwood bedding with enrichment consisting of crinkle cut naturalistic paper strands. Mice were maintained on ad libitum PicoLab Rodent Diet 20 (5053) and water supply with 30-70% environmental humidity and temperature of 20 – 26.2oC. All mice were health checked and monitored daily for food and water intake by trained personnel. Mice of folloing strains were used: FVB/NJ (Jackson Laboratory; Strain#:001800) and CD-1 (Charles River Laboratories; Strain Code: 022), mice between 7-8 weeks of age were used for all crosses, and those between developmental stages E10.5-E15.5 were used across experiments in this study. Sample size selection strategies and scoring criteria were followed based on our experience of performing transgenic mouse assays for ~3000 published enhancer candidates.
Wild animals	n/a
Reporting on sex	Animals of both sexes were included in the experiments. However, we did not consider embryo sex as a variable in our studies since craniofacial development is expected to show minimal differences at these early stages of development.
Field-collected samples	n/a
Ethics oversight	All animal work was reviewed and approved by the Lawrence Berkeley National Laboratory Animal Welfare and Research Committee.

Note that full information on the approval of the study protocol must also be provided in the manuscript.

Plants

Seed stocks	n/a
Novel plant genotypes	n/a
Authentication	n/a

ChIP-seq

Data deposition

- [x] Confirm that both raw and final processed data have been deposited in a public database such as GEO.
- [x] Confirm that you have deposited or provided access to graph files (e.g. BED files) for the called peaks.

Data access links<br>*May remain private before publication.*	https://doi.org/10.25550/3C-4G62 (2023).
Files in database submission	cs18_12612_h3k27ac.fastq.gz<br>LPCS_33016_2C_cs18_face_12676_h3k27ac.fastq.gz<br>cs18_face_12695_h3k27ac.fastq.gz<br>LPCS_33016_2D_cs19_face_12696_h3k27ac.fastq.gz<br>LPCS_081715_1A_cs22_11963_face_h3k27ac.fastq.gz<br>chip1_S2_L001_R1_001.fastq.gz<br>LPCS_021617_1D_cs23_face_12492_h3k27ac.fastq.gz<br>chip1_S2_L001_R2_001.fastq.gz<br>chip1_S2_L002_R1_001.fastq.gz<br>chip1_S2_L002_R2_001.fastq.gz

CS18-12612-H3K27ac.bw
CS18-12676-H3K27ac.bw
CS18-12695-H3K27ac.bw
CS19-12696-H3K27ac.bw
CS22-11963-H3K27ac.bw
CS22-12523r1-H3K27ac.bw
CS23-12492-H3K27ac.bw
CS22-12523r2-H3K27ac.bw
CS18-12612-H3K27ac_peaks-q1.3.broadPeak
CS18-12676-H3K27ac_peaks-q1.3.broadPeak
CS18-12695-H3K27ac_peaks-q1.3.broadPeak
CS19-12696-H3K27ac_peaks-q1.3.broadPeak
CS22-11963-H3K27ac_peaks-q1.3.broadPeak
CS22-12523r1-H3K27ac_peaks-q1.3.broadPeak
CS23-12492-H3K27ac_peaks-q1.3.broadPeak
CS22-12523r2-H3K27ac_peaks-q1.3.broadPeak

Genome browser session
(e.g. UCSC)

Provide a link to an anonymized genome browser session for "Initial submission" and "Revised version" documents only, to enable peer review. Write "no longer applicable" for "Final submission" documents.

Methodology

Replicates

Reported in Methods where applicable.

Sequencing depth

Sample | Total Reads | Uniquely mapped reads | Read length | Read type
CS18_12612_face_h3k27ac | 49,267,494 | 36,896,224 | 50bp | single-end
CS18_12676_face_h3k27ac | 50,479,476 | 38,069,332 | 50bp | single-end
CS18_12695_face_h3k27ac | 41,931,429 | 31,477,603 | 50bp | single-end
CS19_12696_face_h3k27ac | 46,180,716 | 34,607,018 | 50bp | single-end
CS22_12523r1_face_h3K27ac | 50,421,056 | 38,368,562 | 150bp | paired-end
CS22_12523r2_face_h3K27ac | 56,450,756 | 42,619,022 | 150bp | paired-end
CS22_11963_face_h3k27ac | 18,437,751 | 15,447,007 | 50bp | single-end
CS23_12492_face_h3k27ac | 58,582,851 | 45,518,795 | 50bp | single-end

Antibodies

Anti-H3K27ac antibody, Active Motif, Cat# 39133, Lot 01613007.

Peak calling parameters

ChIP-seq data was analyzed using the ENCODE histone ChIP-seq Unary Control Unreplicated pipeline (https://www.encodeproject.org/pipelines/ENCPL841HGV/) implemented at DNAnexus (https://www.dnanexus.com).

Data quality

Sample | Total peaks | Peaks at FDR 5% | Peaks at FDR 5% and Fold enrichment > 5x
CS18_12612_face_h3k27ac | 211,710 | 17,714 | 3,528
CS18_12676_face_h3k27ac | 277,985 | 11,154 | 512
CS18_12695_face_h3k27ac | 178,769 | 21,648 | 4,496
CS19_12696_face_h3k27ac | 177,751 | 11,720 | 1,732
CS22_12523r1_face_h3K27ac | 216,546 | 28,037 | 8,386
CS22_12523r2_face_h3K27ac | 216,721 | 27,146 | 8,725
CS22_11963_face_h3k27ac | 350,498 | 9,870 | 867
CS23_12492_face_h3k27ac | 159,393 | 21,112 | 1,489

Software

Reads were mapped to the human reference genome version hg38 using BWA (v0.7.7) and sorted bam file generated using samtools (v0.1.19). For the ChIP-seq datasets at CS13-15, CS17 and CS2026, publicly available and post-mapped TagAlign files were used. Peak calling was performed using MACS2 (v2.2.4; --broad flag, q-value < 0.05); upon broad peak calling and applying the FDR filter, bed files were combined and merged using bedtools114. A combined peak set was called by merging peaks from all samples, and overlapping peaks for each sample were counted using overlap_peaks.py.
